# Impact of COVID-19 pandemic on asthma exacerbations: Retrospective cohort study of over 500,000 patients in a national English primary care database

**DOI:** 10.1016/j.lanepe.2022.100428

**Published:** 2022-06-15

**Authors:** Syed Ahmar Shah, Jennifer K. Quint, Aziz Sheikh

**Affiliations:** a Asthma UK Centre for Applied Research, Usher Institute, The University of Edinburgh, Edinburgh, UK; b National Heart & Lung Institute, Imperial College London, UK

**Keywords:** Asthma, COVID-19, Asthma Exacerbations, Pandemic

## Abstract

**Background:**

Several countries reported a substantial reduction in asthma exacerbations associated with COVID-19 pandemic-related restrictions. However, it is not known if these early reported declines were short-term and if these have rebounded to pre-pandemic levels following easing of lockdown restrictions.

**Methods:**

We undertook a retrospective, cohort study of all asthma patients in a national primary care database of almost 10 million patients, Optimum Patient Care Database (OPCRD), identified from January 1, 2010, to December 31, 2015, using a previously validated algorithm. We subsequently followed the identified cohort of asthma patients from January 1, 2016, to October 3, 2021, and identified every asthma exacerbation episode with a validated algorithm. To quantify any pandemic-related change in exacerbations, we created a control time-series (mean of 2016-2019) and then compared the change in exacerbation rate in 2020-2021 over quarterly periods when compared with the control period (the pre-pandemic period). We undertook overall and stratified analyses by age group, sex, and English region.

**Findings:**

We identified 100,362 asthma patients (502,669 patient-years) from across England who experienced at least one exacerbation episode (298,390 exacerbation episodes during the entire follow-up). Except for the first quarter of 2020, the exacerbation rates were substantially lower (>25%) during all quarters in 2020-2021 when compared with the rates during 2016-2019 (39.7% (95% Confidence Interval (CI): 34.6, 44.9) in quarter-2, 2020; 46.5% (95%CI: 36.7, 56.4) in quarter-3, 2020; 56.3% (95%CI: 48.7, 63.9) in quarter-4, 2020; 63.2% (95%CI: 53.9, 72.5) in quarter-1, 2021; 57.7% (95%CI: 52.9, 62.4) in quarter-2, 2021; 53.3% (95%CI: 43.8, 62.8) in quarter-3, 2021).

**Interpretation:**

There was a substantial and persistent reduction in asthma exacerbations across England over the first 18 months after the first lockdown. This is unlikely to be adequately explained by changes in health-seeking behaviour, pandemic-related healthcare service disruption, or any air-quality improvements.

**Funding:**

Asthma UK, Health Data Research UK (HDR UK), Medical Research Council (MRC), National Institute for Health Research (NIHR).

**Research in context**

**Evidence before this study**

We searched PubMed and medRxiv on November 21, 2021, using the query “asthma AND (exacerbation OR attack) AND (SARS-CoV-2 OR COVID-19 OR pandemic OR lockdown)” for all studies published since January 1, 2020, with no language restrictions and limiting PubMed search span to [Title/Abstract]. Of the 122 search results (48 on medRxiv, and 74 on PubMed), after screening each study's abstract we identified 47 potentially relevant studies. We also identified two additional studies that we deemed relevant.

Overall, 20 studies investigated asthma exacerbation patterns during the pandemic, and they all reported substantial reductions in exacerbation rates. Four studies used self-reported data, and 16 studies used routinely collected data. However, most of the studies covered a small geographical area, often from a single hospital and 10 studies focused on paediatric patients only. The five relatively large, country-wide studies that used routinely collected data were from Scotland and Wales (Davies et al.), Hong Kong (Chan et al.), and Japan (Bun et al.) using hospital data, and from England (our previous study) and the UK (Mansfield et al.) using primary care recorded data. All these relatively large sample size studies had a short follow-up with the longest one reported in our previous study (until August 2020). Even amongst all 20 studies, the follow-up time was mostly short, and they could only assess the short-term impact of the pandemic when various restrictions were freshly imposed and when there were no COVID-19 vaccinations. The only exception was a study from Dublin (Quintyne et al.) that assessed the pandemic impact on asthma exacerbations until February 2021.

In summary, several studies from various countries have reported a substantial reduction in asthma exacerbations that were attributed to pandemic-related restrictions in 2020. However, it was not clear if the early reported, substantial reductions were short-term and if there has been a rebound to pre-pandemic levels with easing of restrictions.

**Added value of this study**

To our knowledge, this is the longest and one of the largest studies to assess the impact of the pandemic on asthma exacerbations. Previous short-term studies offered various hypotheses to explain the substantial reduction in asthma exacerbations during the pandemic, such as improved air quality, change in health-seeking behaviours due to fear of COVID-19, healthcare disruption, and reduced exposure to other viruses.

By demonstrating substantial and sustained reduction in asthma exacerbations over 18 months (since the first lockdown), during periods with varied pandemic-related public health measures, this analysis suggests that change in health-seeking behaviour due to fear of COVID-19, healthcare disruption and improved air quality are unlikely to explain a consistent and sustained reduction in asthma exacerbations.

**Implications of all the available evidence**

A substantial and sustained reduction in asthma exacerbations is possible with various primary and public health measures. Further research is required to design and develop pragmatic interventions to ensure that the low rate of asthma exacerbations can persist beyond the COVID-19 pandemic.

Alt-text: Unlabelled box

## Introduction

Several countries reported a substantial reduction in asthma exacerbations during the on-going COVID-19 pandemic.^1^, ^2^, ^3^, ^4^, ^5^, ^6^, ^7^, ^8^, ^9^, ^10^, ^11^ These early studies, however, focused on the short-term impact of emergency restrictions imposed by several countries after the World Health Organization (WHO) declared a pandemic in March 2020^12^^,^^13^; little is however known about longer-term impact.

Since the pandemic, the United Kingdom (UK) government has thrice imposed strict emergency restrictions (i.e., national lockdowns) with the first one in March 2020.^13^ In addition, the government also introduced a shielding list of people deemed to be at a high risk of developing serious COVID-19 outcomes who were initially advised to stay at home; this list included patients with asthma.^14^ We previously investigated the short-term impact of the first UK-wide lockdown on asthma exacerbations in England and reported a substantial reduction immediately after the first lockdown.^10^ Beginning from 2021, as wide-spread vaccinations were rolled out across England, pandemic-related restrictions were gradually eased, and nearly all restrictions were lifted on July 19, 2021.^15^ The shielding list programme also effectively ended on July 19, 2021 when people on the list were advised to follow the same guidance as the rest of the population.^16^ Nevertheless, the number of daily reported Severe Acute Respiratory Syndrome Coronavirus-2 (SARS-CoV-2) infections in England have remained high since then with a seven-day average of at least 20,000 daily cases (appendix p2). Consequently, there is a need to understand how people with asthma have been impacted with regards to exacerbations in the context of minimal pandemic-related restrictions during a period of sustained high SARS-CoV-2 infection levels to ascertain if the early reported drop in exacerbation rates was short-term and if there has been a rebound to pre-pandemic levels with easing of restrictions.

In this study, we aimed to investigate the impact of the pandemic, and the associated measures that have ranged from strict, country-wide lockdown to minimal restrictions, on asthma exacerbations over 18 months since the first national lockdown.

## Methods

### Data source and setting

The Optimum Patient Care (OPC) network collects anonymised clinical data from General Practitioner (GP) practices from across the UK. This dataset is fully anonymised and hosted in a dedicated data repository called the Optimum Patient Care Research Database (OPCRD). Access to OPCRD is regulated and available to bona fide researchers subject to licensing agreements and study-specific permissions.

OPCRD has been used in several epidemiological, pharmaceutical, and other observational clinical studies (https://opcrd.co.uk/).^10^ At the time of cohort identification, the OPCRD consisted of almost 10 million patients from 792 practices. The OPCRD database contains diverse types of data recorded for a given patient such as demographics, medical events (diagnoses, symptoms), prescriptions and therapies, and referrals. For this study, we were provided access to the database (a Microsoft SQL database) via a secure connection using OPC's virtual private network (VPN).

### Ethics approvals and permissions

The OPCRD has an existing NHS Research Authority ethics approval for the use of routinely collected data for research (REC Ref:15/EM/150). The Anonymized Data Ethics and Protocol Transparency (ADEPT) Secretariat oversee the use of OPCRD and grant project-specific approvals. To be able to use the OPCRD for this project, we obtained ADEPT approval (reference number:ADEPT1020a).

### Study design and population

We designed a retrospective cohort study and identified all asthma patients through a validated algorithm^17^ from the entire database for the period January 1, 2010, to December 31, 2015. The algorithm was based on the presence of any of 121 Read codes (version 2/3) deemed to be associated with asthma (appendix pp.10-11). From the identified asthma patients, we then restricted our cohort to those who experienced at least one exacerbation episode during the follow-up period. The follow-up period was over 69 months, from January 2, 2016, to October 3, 2021.

### Ascertainment of outcome

The primary outcome, computed for every week during follow-up, was the rate of asthma exacerbation. This rate was computed as the total number of episodes per 100 patient-years. An asthma exacerbation was based on the definition of American Thoracic Society (ATS)/European Respiratory Society (ERS) Task Force definition, previously validated in OPCRD.^18^ A patient was deemed to have experienced an exacerbation episode if one of the following occurred in a given assessment period: i) an asthma-related Accident & Emergency (A&E) visit; ii) a hospital admission due to asthma; or iii) prescription of oral corticosteroids (OCS) with evidence of respiratory review within two weeks.^19^ To further ensure that we did not miss any asthma-related hospitalisation episodes, we identified additional hospital admission codes and then considered a patient to have experienced an exacerbation episode if any of the 121 asthma-specific codes were recorded on the day of hospitalisation (see appendix pp 10-11 for all relevant codes). For a given assessment period (defined as a week in this study) for each patient, asthma exacerbation was treated as a binary variable. This meant that, in each week, a patient would either have an exacerbation episode or remain exacerbation-free.

### Data analysis

To compute the exacerbation rate, we first counted the total number of exacerbation episodes in each week, and then divided it by the number of patients in the cohort in that week. This gave us the exacerbation rate as the number of episodes per patient-week. We then converted this rate into number of episodes per 100 patient-years. We subsequently plotted the exacerbation rate (updated weekly) during the entire follow-up to visually assess any temporal trends and any impact during the pandemic. The exacerbation rate for a given week corresponds to the rate observed over the following seven days starting from a given week's beginning date.

To facilitate statistical comparison between the pandemic and the pre-pandemic period, we divided the entire follow-up into yearly quarters (i.e.,13 weeks). For each quarter, we computed the mean of the 13 weekly exacerbation rates (Figure S2 in appendix p.3) with 95% confidence interval (CI; estimated using 2-tailed t-distribution). We constructed a control time-series from the quarterly exacerbation rates during the pre-pandemic period (2016-2019). The exacerbation rate in each quarter in the control time-series was equal to the mean of corresponding quarterly exacerbation rates during the pre-pandemic period (Figure S3 in appendix p.4). We computed the difference in mean, the 95% CI using the Welch's 2-sample t-test (modified t-test that does not assume equal variances of the two comparison groups) and the percentage difference in the exacerbation rate between a given quarter in 2020-2021 and the corresponding rate during 2016-2019 using the control time-series.

In addition to overall analysis, we carried out stratified analyses. We independently stratified our cohort by sex (males, females), age (0-5, 6-17, 18-54, 55+), and location (East England, East Midlands, London, North East, North West, South East, South West, West Midlands, Yorkshire and the Humber). We only had access to year of birth. We approximated each patient's age by assuming them to be born mid-year (July 1). For age-based stratification, we used the age of each patient on January 1, 2016 (the start date of the follow-up period). We did not have individual-level location data. We therefore used the postcode information of each GP practice to infer location of each patient.

We anticipated attrition of participants over the long follow-up period of almost six years. Possible reasons for this include patients moving out from the area, patient's asthma resolving and the patient having no further contact with the GP, and patients dying. To investigate if there was any difference between the subset of patients who remained and those who dropped out over time, we undertook additional sensitivity analysis by restricting the cohort to only those who stayed until at the last quarter during the follow-up.

Lastly, we undertook additional sensitivity analysis to assess the impact of some patients in the cohort having COPD in addition to asthma. We restricted the cohort to those aged 34 or under at the start date of the follow-up. The cut-off of 34 at the start of the follow-up ensured that all patients in the restricted cohort were under 40 years at the study end date.

All analyses were undertaken in R (version 3.6.2) using RStudio (version 1.4.1717). We used the tidyverse packages for data wrangling (dplyr) and plotting (ggplot2). We used the lubridate package for date manipulation, and the RODBC library to remotely connect to the OPCRD database. “STROBE” and “RECORD” were used to aid transparent reporting.

### Role of the funding source

The funders did not play any role in the study design, data collection, data analysis, interpretation or in the writing of this report.

## Results

### Study cohort demographics

**Figure 1:**
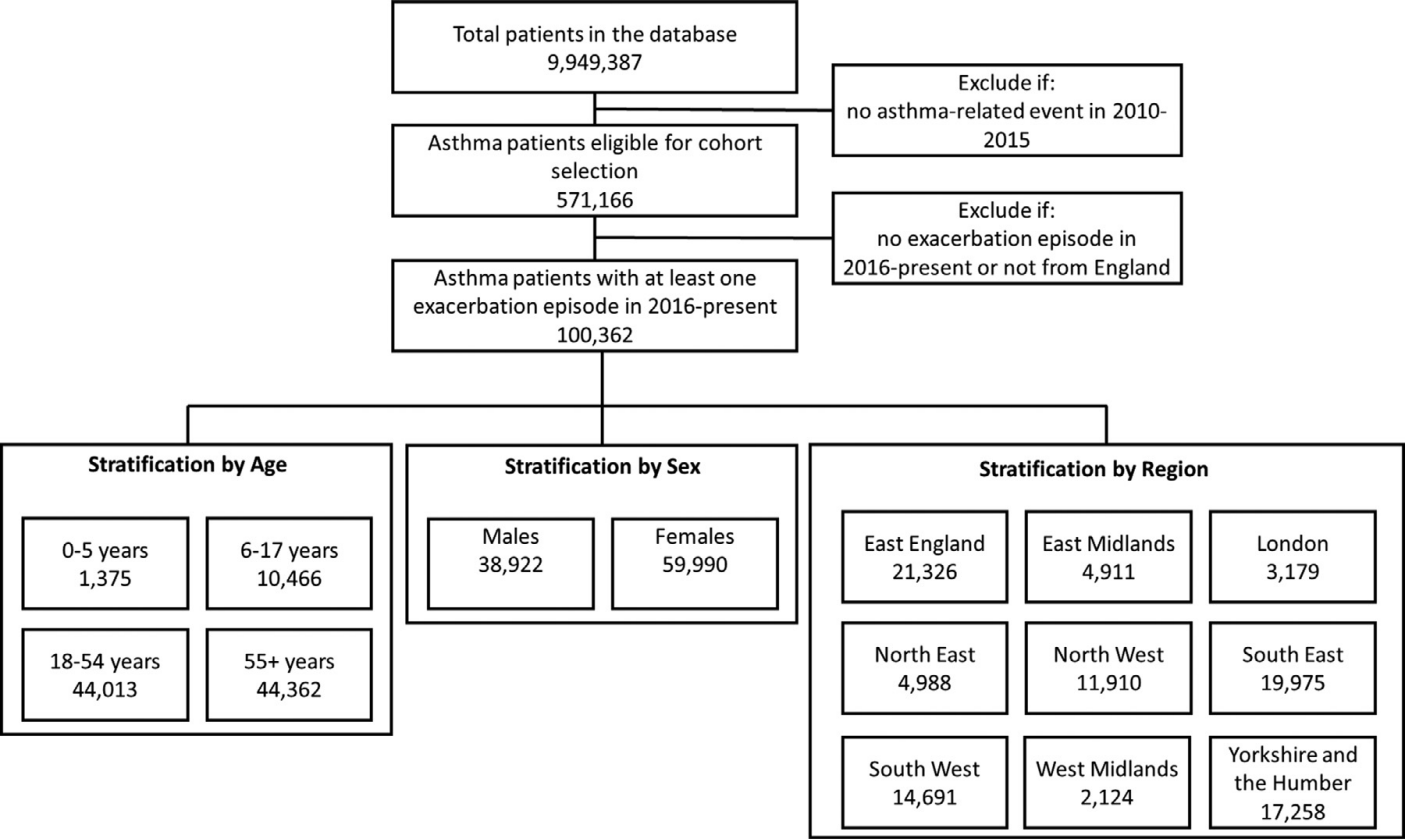
Flow diagram of the patients in the study from OPCRD, with stratification by age, sex, and region. Note that there were 146 patients (0.1%) with missing year of birth information, and 1450 patients (1.4%) with sex information missing.

Out of the 9,949,387 patients in the database, we included 571,166 (giving an asthma prevalence of 5.7%) eligible asthma patients. There were 100,362 (17.6%) patients with at least one exacerbation episode during the follow-up period (i.e., January 1, 2016 – September 27, 2021). The total follow-up period in the study was 502,669 patient-years with a mean follow-up of 5.01 years per patient (Figure 1 shows study flow diagram).

Stratified by age, most patients were either 18-54 (44,013;43.9%) or ≥55years (44,362;44.2%). The number of young patients was relatively small (1375;1.4%). Most of the young patients were 3-5 years old (appendix p.12). A small minority of patients (146;0.1%) did not have birth year recorded in the database and were therefore excluded during age stratification.

Stratified by sex, there were substantially more females (59,990;59.8%) than males (38,922;38.8%). A small minority of patients (1450;1.4%) did not have sex information recorded in the database and were therefore excluded during sex stratification. Stratified by region, a large proportion of patients were from East England, South East, and Yorkshire and the Humber.

When further stratified by both age and sex, there were more males than females in the younger age groups (0-5: 65.1% vs 34.9%; 6-17: 57.1% vs 42.9%) but a substantially greater number of females than males in the older age groups (18-54: 36.2% vs 63.8%; 55+: 37.5% vs 62.5%, appendix p.11). While we were unable to identify any existing previous study that reported prevalence of asthma patients amongst those who had at least one exacerbation during a specific study period, the overall pattern that we observed in our data (more males than females with asthma who have an exacerbation amongst younger age groups, and more females than males with asthma who have exacerbation amongst older age groups) is corroborated by previous studies in the UK^20^ and elsewhere^21^^,^^22^ that have reported similar patterns.

### Exacerbations during follow-up

**Figure 2:**
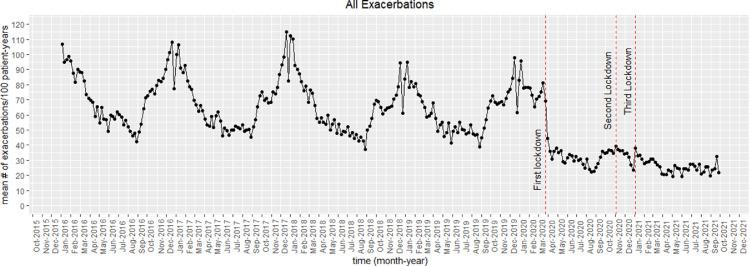
Mean exacerbations rate (number of exacerbations per 100 patient-years) of all asthma patients in the study cohort during the follow-up (January 2016–September 2021).

There were 298,390 exacerbation episodes experienced by the 100,362 patients during the follow-up period. Figure 2 shows the weekly mean exacerbation rate (total number of exacerbation episodes per 100 patient-years) during the entire follow-up period. Before the pandemic (2016-2019), the mean exacerbation rate followed a similar seasonal pattern with a peak during winters (December-January) followed by a gradual drop to reach a trough during the summer period (June-July). This was followed by a gradual increase from September, likely in tandem with schools re-opening post-summer break. The big single week drop observed towards the end of December corresponded to restricted primary care timings during the Christmas period. During the beginning of the pandemic, there was a significant and substantial decrease in exacerbation rates after the imposition of the first national lockdown in March 2020. Like the seasonal pattern observed during the previous years, there was a gradual increase in exacerbation rates from August 2020 until the imposition of the second lockdown. Overall, though, the exacerbation rates were consistently substantially lower since the pandemic began, compared to the rates during pre-pandemic years.

Table 1 provides the mean exacerbation rate during follow-up in quarterly intervals (13 weeks), the total number of patients at the beginning of the respective interval, and the total number of exacerbation episodes. Until the first quarter of 2020, the exacerbation rates ranged from 48.7 (observed in weeks 27-39 in 2018) to 88.9 (observed in weeks 1-3 in 2016). Since the beginning of the pandemic, the exacerbation rates have ranged from 23.2 (observed in weeks 14-26 in 2021) to 34.1 (observed in weeks 40-52 in 2020).

**Table 1:** The mean exacerbation rates throughout the follow-up period of all the patients in the cohort, with the periods divided into quarter periods (13 weeks). The weeks refer to ISO week, the number of patients refer to the total number of patients at the beginning of the respective period, and the number of episodes refer to the total number of exacerbation episodes in the respective period.

Period	Number of Patients	Number of Episodes	Exacerbation Rate (95% CI)
2016 (weeks 1-13)	100,362	22,271	88.9 (83.4 - 94.4)
2016 (weeks 14-26)	100,045	15,019	60.2 (57.1 - 63.3)
2016 (weeks 27-39)	99,473	13,455	54.2 (49.8 - 58.5)
2016 (weeks 40-52)	99,137	20,956	84.7 (78.6 - 90.7)
2017 (weeks 1-13)	98,893	19,785	80.3 (72.3 - 88.2)
2017 (weeks 14-26)	98,362	13,098	53.3 (50.7 - 56.0)
2017 (weeks 27-39)	98,081	13,191	53.9 (49.9 - 57.9)
2017 (weeks 40-52)	97,769	19,773	81.1 (73.6 - 88.7)
2018 (weeks 1-13)	97,177	20,004	82.5 (73.8 - 91.2)
2018 (weeks 14-26)	96,655	12,780	53.0 (50.8 - 55.2)
2018 (weeks 27-39)	95,890	11,623	48.7 (44.6 - 52.8)
2018 (weeks 40-53)	95,033	17,851	70.2 (65.3 - 75.2)
2019 (weeks 1-13)	93,880	16,833	72.3 (66.6 - 78.0)
2019 (weeks 14-26)	91,680	11,540	50.7 (48.3 - 53.1)
2019 (weeks 27-39)	90,078	11,342	51.2 (46.7 - 55.6)
2019 (weeks 40-52)	87,396	15,567	73.8 (68.5 - 79.0)
2020 (weeks 1-13)	79,414	14,307	73.2 (67.0 - 79.5)
2020 (weeks 14-26)	76,870	6207	32.7 (31.0 - 34.4)
2020 (weeks 27-39)	74,668	5183	28.0 (25.6 - 30.3)
2020 (weeks 40-52)	73,243	6043	33.8 (31.5 - 36.2)
2021 (weeks 1-13)	68,812	4884	29.4 (27.1 - 31.6)
2021 (weeks 14-26)	65,194	3493	23.0 (21.6 - 24.4)
2021 (weeks 27-39)	58,776	3185	24.4 (22.6 - 26.3)

CI: Confidence Interval.

The mean exacerbation rates during the entire follow-up stratified by sex, age, and region broadly follow a similar pattern (appendix, pp 5-7). There was a strong seasonality pattern during the pre-pandemic period, and there was a consistent and substantial reduction in exacerbation rates during the pandemic.

The most common hospital-related code used in our study was “663m.” which is defined as “Asthma A&E attendance since last visit”. Due to lack of linkage with a secondary care data source (such as Hospital Episode Statistics), we were unable to determine with certainty if a given previous attendance to A&E due to asthma got resolved on the day of attendance or if it required hospitalisation. We have, therefore, stratified the exacerbation type by the clinical setting where it got resolved: primary care, and hospital (includes both A&E attendance and admissions). Figure S9 (appendix p.7) shows the overall exacerbation rates during the follow-up when stratified by clinical setting. Even before the pandemic, most asthma exacerbation episodes were resolved within primary care. In our dataset, 263,120 exacerbation episodes (88.2%) were resolved in primary care and 35,270 (11.8%) were resolved in hospital. It is evident that the overall asthma exacerbation trend during the follow-up is primarily driven by exacerbations that get resolved within primary care.

### Statistical comparison of exacerbation rates between 2016-2019 and 2020-2021

**Figure 3:**
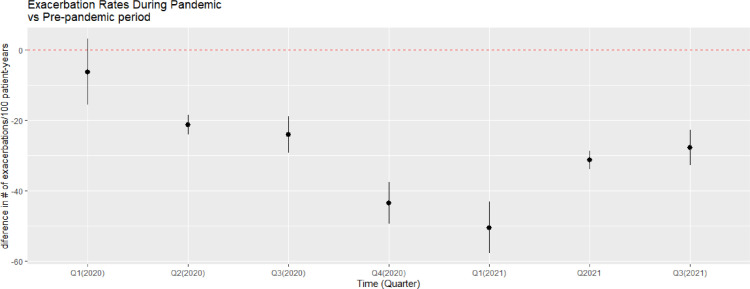
Difference in mean exacerbations rate (number of exacerbations per 100 patient-years) between the pandemic period (2020-2021) and the pre-pandemic period (mean of 2016-2019) by quaterly periods.

Overall, the mean exacerbation rate was not substantially (<25%) different during the first quarter of 2020 compared with the control period (mean of 2016-2019). However, the exacerbation rates were substantially lower (> 25%) during all remaining time intervals in 2020-2021 when compared with the rates during 2016-2019 (39.7% in quarter 2,2020; 46.5% in quarter 3,2020; 56.3% in quarter 4,2020; 63.2% in quarter 1,2021; 57.7% in quarter 2,2021; 53.3% in quarter 3,2021; Figure 3).

Table 2 summarises the change in mean exacerbation rate, the percentage change, and the associated 95% CI in 2020 (quarters 1-4), and 2021 (quarters 1-3) compared with the mean exacerbation rates during the corresponding period in 2016-2019. For every stratum considered, the difference in mean exacerbation rates during the first quarter in 2020 compared to 2016-2019 was not substantially different (except for the 0-5 group which showed a reduction of 43.6%). However, the mean exacerbation rates dropped substantially starting from quarter 2,2020 and since quarter 3,2020, the drop in mean exacerbation rates have been substantial with at least 25% drop in every stratum considered.

**Table 2:** Difference in mean exacerbation rate between the control period (mean rate during 2016-2019) and 2020-2021. The exacerbation rates are the total number of episodes per 100 patient-years. The percentage change is relative to the rate during the control period. The 2020-2021 period is divided into equal, 13-week intervals.

Cohort	Mean exacerbations rate compared to the rate during the control period (2016-2019); Percentage change compared to the control period (%); 95% Confidence Interval						
	Quarter 1 (2020)	Quarter 2 (2020)	Quarter 3 (2020)	Quarter 4 (2020)	Quarter 1 (2021)	Quarter 2 (2021)	Quarter 3 (2021)
All (*n* = 100,362)	-6.6; -8.3% (-16.0, 2.8)	-21.6; -39.7% (-24.3, -18.8)	-24.3; -46.5% (-29.5, -19.2)	-43.6; -56.3% (-49.5, -37.7)	-50.5; -63.2% (-57.9, -43.1)	-31.3; -57.7% (-33.9, -28.7)	-27.9; -53.3% (-32.9, -22.9)
**Stratification by Sex**							
Males (*n* = 38,922)	-6.8; -9.2% (-16.5, 2.9)	-20.5; -40.5% (-23.1, -18.0)	-24.7; -50.3% (-29.7, -19.7)	-40.5; -57.5% (-46.1, -35.0)	-49.0; -65.9% (-55.8, -42.1)	-31.7; -62.6% (-34.2, -29.3)	-28.0; -57.0% (-32.7, -23.3)
Females (*n* = 59,990)	-5.6; -6.8% (-15.4, 4.1)	-21.6; -38.2% (-24.9, -18.3)	-23.5; -43.3% (-29.1, -18.0)	-45.3; -55.0% (-51.6, -38.9)	-51.0; -61.0% (-58.8, -43.1)	-30.0; -52.9% (-33.1, -26.8)	-26.6; -48.9% (-32.0, -21.2)
**Stratification by Age**							
0-5 (*n* = 1375)	-30.7; -43.6% (-45.8, -15.6)	-38.2; -81.2% (-45.9, -30.4)	-24.5; -57.0% (-39.9, -9.1)	-58.1; -75.5% (-69.0, -47.2)	-56.3; -79.9% (-67.4, -45.2)	-38.5; -81.9% (-44.8, -32.1)	-33.6; -78.1% (-48.2, -19.0)
6-17 (*n* = 10,466)	-5.3; -10.5% (-13.0, 2.3)	-21.7; -59.1% (-24.7, -18.7)	-22.1; -57.1% (-32.6, -11.6)	-33.2; -61.4% (-39.3, -27.2)	-37.3; -73.1% (-42.3, -32.3)	-25.8; -70.4% (-28.7, -23.0)	-22.5; -58.2% (-32.3, -12.8)
18-54 (*n* = 44,013)	4.7; 7.0% (-4.8, 14.2)	-10.4; -23.3% (-14.3, -6.5)	-17.8; -39.3% (-23.3, -12.3)	-35.5; -51.1% (-40.3, -30.7)	-35.2; -52.5% (-42.4, -28.0)	-21.9; -49.0% (-24.5, -19.3)	-20.8; -45.9% (-26.0, -15.6)
55+ (*n* = 44,362)	-16.0; -16.0% (-29.6, -2.4)	-31.4; -45.9% (-35.0, -27.7)	-30.5; -48.5% (-34.7, -26.4)	-53.1; -58.1% (-61.5, -44.8)	-68.2; -68.2% (-77.6, -58.9)	-41.1; -60.0% (-44.7, -37.4)	-35.4; -56.3% (-39.4, -31.4)
**Stratification by Region**							
East England (*n* = 21,326)	-17.4; -21.2% (-27.4, -7.4)	-30.5; -55.5% (-34.4, -26.5)	-22.8; -46.7% (-27.9, -17.6)	-44.3; -60.1% (-50.4, -38.2)	-55.2; -67.4% (-62.3, -48.1)	-32.5; -59.3% (-36.8, -28.3)	-21.2; -43.4% (-26.4, -16.0)
East Midlands (*n* = 4911)	5.6; 8.2% (-5.2, 16.3)	-23.9; -49.0% (-28.2, -19.6)	-29.0; -60.7% (-36.3, -21.6)	-46.1; -64.0% (-52.2, -40.0)	-46.8; -68.8 (-52.8, -40.7)	-25.5; -52.3% (-30.1, -21.0)	-22.8; -47.7% (-29.9, -15.6)
London (*n* = 3179)	3.7; 6.5% (-5.3, 12.8)	-10.1; -21.1% (-17.9, -2.2)	-16.0; -33.7% (-25.9, -6.0)	-37.4; -49.5% (-47.9, -26.9)	-32.3; -56.3% (-37.6, -27.0)	-30.7; -64.2% (-35.0, -26.3)	-25.0; -52.7% (-32.8, -17.3)
North East (*n* = 4988)	2.0; 2.3% (-11.7, 15.7)	-8.5; -14.2% (-18.2, 1.2)	-20.0; -33.6% (-27.8, -12.2)	-34.1; -38.6% (-42.5, -25.6)	-44.2; -51.2% (-55.9, -32.6)	-29.3; -48.7% (-33.6, -24.9)	-25.3; -42.5% (-31.3, -19.3)
North West (*n* = 11,910)	10.1; 11.8% (-1.5, 21.7)	-10.3; -17.6% (-17.9, -2.6)	-24.9; -42.4% (-30.2, -19.5)	-28.3; -33.3% (-36.9, -19.8)	-36.5; -42.4% (-46.7, -26.4)	-28.4; -48.8% (-32.6, -24.2)	-28.5; -48.7% (-34.5, -22.6)
South East (*n* = 19,975)	-9.2; -11.4% (-19.5, 1.2)	-23.6; -43.8% (-27.7, -19.4)	-26.3; -49.9% (-33.6, -19.0)	-50.0; -64.2% (-56.4, -43.6)	-50.4; -62.7% (-58.9, -41.9)	-34.4; -64.0% (-37.3, -31.5)	-34.5; -65.4% (-39.3, -29.6)
South West (*n* = 14,691)	-6.5; -8.2% (-18.3, 5.3)	-21.3; -41.4% (-24.1, -18.4)	-24.5; -51.0% (-29.9, -19.2)	-49.4; -67.4% (-57.2, -41.5)	-56.8; -71.8% (-64.6, -49.0)	-33.2; -64.7% (-36.0, -30.4)	-28.2; -58.5% (-33.6, -22.7)
West Midlands (*n* = 2124)	20.9; 23.1% (0.4, 41.5)	-6.4; -10.3% (-23.2, 10.4)	-20.7; -35.2% (-30.6, -10.8)	-43.3; -50.2% (-52.5, -34.1)	-61.8; -68.2% (-78.3, -45.2)	-25.2; -40.8% (-33.6, -16.8)	-32.0; -54.2% (-44.6, -19.4)
Yorkshire and the Humber (*n* = 17,258)	-7.6; -9.8% (-16.5, 1.4)	-20.5; -38.2% (-24.0, -17.0)	-23.5; -43.3% (-30.3, -16.7)	-40.8; -52.6% (-47.3, -34.3)	-49.6; -64.4% (-56.9, -42.2)	-27.7; -51.6% (-31.3, -24.1)	-27.4; -50.4% (-33.5, -21.3)
**Stratification by Primary/Secondary Care**							
Primary Care	-13.2; -27.3% (-21.6, -4.8)	-24.9; -51.4% (-27.6, -22.2)	-22.1; -49.2% (-27.3, -16.9)	-42.5; -61.5% (-48.6, -36.3)	-51.1; -70.1% (-57.9, -44.3)	-26.0; -53.7% (-28.7, -23.3)	-21.2; -47.2% (-25.8, -16.6)
Secondary Care^a^	6.6 (4.9, 8.2)	3.4 (2.0, 4.7)	-2.2 (-4.2, -0.3)	-1.2 (-3.1, 0.8)	0.6 (-0.6, 1.8)	-5.3 (-5.8, -4.8)	-6.7 (-7.1, -6.2)

a Only the change in rate is shown since the secondary care-related exacerbation rate is small and substantially lower than primary care-related exacerbation rate.

Stratified by sex, the mean exacerbation rate dropped by up to 65.9% (quarter1,2021) for males, and 61.0% for females (quarter1,2021). Stratified by age, the mean exacerbation rate dropped by up to 81.9% for 0-5 (quarter2,2021), 73.1% for 6-17 (quarter1,2021), 52.5% for 18-54 (quarter1,2021), and 68.2% for 55+ (quarter1,2021). Stratified by region, the mean exacerbation rate dropped by up to 67.4% for East England (quarter1,2021), 68.8% for East Midlands (quarter1,2021), 64.2% for London (quarter2,2021), 51.2% for North East (quarter1,2021), 48.8% for North West (quarter2,2021), 65.4% for South East (quarter3,2021), 71.8% for South West (quarter1,2021), 68.2% for West Midlands (quarter1,2021), and 64.4% for Yorkshire and the Humber (quarter1,2021). Stratified by whether an exacerbation episode was resolved within primary care or whether it required a hospital visit (secondary care), the change in secondary care-based exacerbation rates is small, ranging from 3.4 (95% CI: 2, 4.7) to -6.7 (95% CI: -7.1, -6.2) episodes per 100 patient-years. However, the reduction in exacerbations that were resolved within primary care is substantial, ranging from -51.1 (95% CI: -57.9, -44.3) to -21.2 (95% CI: -25.8, -16.6) episodes per 100 patient-years.

### Sensitivity Analyses

Figures S12 and S13 (appendix p.9) plot the exacerbation rates over time when the cohort is restricted to the: 58,776 patients who stayed in the cohort until at the last quarter during the follow-up; 27,095 patients who were aged 34 or under at the start of the follow-up period. The overall pattern in these sub-cohorts during the follow-up was similar to the pattern of the entire cohort: strong seasonal pattern before the pandemic peaking in winters (December-January 2016-2019), gradually dropping and reaching a trough in the summers (May-June 2016-2019), gradually rising again from September 2016-2019, substantial reduction beginning from the first lockdown period (March 2020) and the large drop during the pandemic sustaining until the study end date of October 3, 2021. It is evident from the figures that there is no systematic difference between taking the entire cohort and restricting the cohort to only those who remained until the end of the study, or to those aged 34 or under at the start of the follow-up period.

## Discussion

This large nationwide analysis has found substantial reductions (up to 63% overall) in asthma exacerbations over 18 months since the imposition of the first lockdown in March 2020. These reductions were observed for both males and females, all age groups, and all regions across England and all periods studied.

In addition to our previous study suggesting a substantial, short-term reduction in asthma exacerbations at the beginning of the pandemic,^10^ several studies have reported similar findings, albeit over a much shorter timeframe (see appendix pp 12-14 for complete list).^1^, ^2^, ^3^, ^4^, ^5^, ^6^, ^7^, ^8^, ^9^^,^^11^ Most previous studies covered a small geographic area, often from a single hospital with a relatively small sample size. However, there were notable exceptions. A few studies that were large, multi-centered, and used routinely collected data included studies from Scotland, Wales, and Japan using hospital data^5^^,^^7^ and another UK-wide study using primary care data.^23^ In addition, there were two large studies using self-reported data, one from the US,^3^ and a global study with over 1500 children recruited from 15 countries.^24^ All the afore-mentioned studies reported a substantial reduction in asthma exacerbations after the pandemic onset. However, these studies only assessed the short-term impact (less than six months) of the pandemic with the longest follow-up until July 2020.^23^ Consequently, these studies could not ascertain whether the reduction observed was primarily due to immediate lockdown measures with a likely rebound effect after most COVID-related restrictions were lifted or whether the reduction persisted long-term.

Most previous studies that used routinely collected healthcare data leveraged secondary-care data only. Consequently, these studies were only able to assess the impact of the pandemic on severe asthma exacerbations that required a hospital visit. However, the majority of UK asthma patients are treated in primary care.^25^ In addition to any primary care interactions, the UK primary care records also include any A&E attendances and hospitalisation episodes. By leveraging UK primary care databases therefore, we were able to capture exacerbation episodes that may require hospital visits as well as those that get resolved within primary care. This facilitates a more comprehensive assessment of the pandemic on the wider asthma population. Apart from our previous report,^10^ the only other study that leveraged UK primary care data was reported by Mansfield et al.,^23^ that assessed short-term impacts.

Previous studies have suggested several factors to explain the substantial reduction in asthma exacerbations during the pandemic. These included a change in care-seeking behaviour with patients more likely to avoid contacting visiting healthcare facilities due to fear of contracting COVID-19,^26^ disruption to healthcare services,^23^ reduction in air pollution,^11^ a reduced circulation of other respiratory viruses due to reduced mobility during the pandemic, improved hygiene and protection due to measures such as frequent hand-washing, sanitising, and use of face-masks, and improved self-management including high medication adherence rate.^2^^,^^3^^,^^5^, ^6^, ^7^^,^^9^^,^^10^^,^^24^

While early studies suggested that some people might avoid seeking healthcare facilities due to fear of COVID-19,^26^ the National Health Service (NHS England) mounted a public information campaign beginning in 2020 to persuade people to use healthcare services if required.^27^ In addition, all vulnerable people (including a subset of those with asthma) were prioritised for receiving COVID-19 vaccines after the roll-out began in December 2020. Consequently, we think it is most unlikely that avoiding healthcare services is a key factor. This is further corroborated by Sykes et al.^6^ who found a clear reduction in proportion of patients with high severity of disease in those admitted with asthma (which should have instead increased with delayed presentation). Further, another study reported substantial reductions in asthma exacerbations amongst 1,178 patients recruited from across the US as measured by remote, self-reported, monthly ‘Asthma Exacerbation Questionnaire’ thereby suggesting genuine reductions.^3^

Similarly, healthcare disruption is also unlikely to be a key factor since many pandemic-related restrictions were eased during the long follow-up, first in summers 2020, and then from July 19, 2021 after widespread vaccination coverage, in an attempt to mitigate pandemic-related disruptions.^13^ To further demonstrate that the sustained reduction in asthma exacerbations were not likely due to difficulty in accessing primary care, we investigated how the pattern of monthly consultations changed over time in England. We used publicly available data released by NHS Digital on activity and usage of GP appointments.^28^ The pattern of total monthly GP appointments clearly demonstrate that there was a substantial reduction in the total number of appointments in England beginning from March 2020 (when the first lockdown was imposed). However, the total number of GP appointments gradually returned to pre-pandemic levels within six months (by September 2020, Figure S11, appendix p.8). This is further corroborated by another study that found substantial reduction in paediatric visits to hospital after pandemic onset returned to pre-pandemic levels by June/July 2020 except for asthma exacerbations.^1^ We also believe that improved air quality was unlikely to be a key factor since outdoor air pollution levels have gradually returned to pre-pandemic levels, but the drop in exacerbation rates have persisted longer-term.^1^ This is further corroborated by other studies suggesting that exposure to viruses is a more important factor than pollution levels.^4^^,^^8^

While it is not possible to ascertain the role (and extent) of any improved self-management, this could have contributed to the reduction observed since patients with chronic conditions were more likely to adhere to pandemic-related restrictions as opposed to those without any chronic conditions.^29^ There were early suggestions that, amongst asthma patients, medication adherence improved during the pandemic.^30^ The evidence to date is, however, conflicting. A recent study from England suggested only a modest increase in adherence to Inhaled Corticosteroids (ICS) medication in the first year of the pandemic primarily driven by increased prescription in March 2020.^31^ Another study from England suggests that medication use declined during the first eight months of the pandemic.^32^ Studies from the US have also suggested decreased adherence to controller medications amongst patients with asthma for both children^33^ and adults^34^ during the pandemic. Even if the observed reductions were due to improved self-management including high medication adherence during the pandemic, more plausible underlying factors are the various non-pharmacological interventions during the pandemic that led to reduced social interactions and the associated reduced risks of contracting viral infections. This is because no new pharmacological interventions (beyond what was available before the pandemic) were introduced during the pandemic and advice on asthma management and asthma exacerbations have remained unchanged.^35^ These hypotheses all need more detailed investigation.

Overall, the most likely explanation for the substantial, persistent drop in exacerbation rates seen across England is reduced exposure to respiratory viruses such as rhinoviruses that are common triggers of asthma exacerbations.^36^ This reduction likely came about due to widespread adoption of pandemic-related measures such as mask-wearing, social distancing, and improved hygiene including frequent handwashing and cleaning of public places. This is further corroborated by several previous studies that suggest that exposure to common respiratory viruses is a major trigger of asthma exacerbations.^37^ In addition, a recent study from Singapore reported a substantial reduction in asthma admissions with viral infections over eight months that coincided with pandemic-related health measures.^38^ While exposure to other common respiratory viruses has reduced during the pandemic, it is reasonable to expect a relatively higher risk of exposure to SARS-CoV-2 during the pandemic. However, increasing number of studies have suggested that SARS-CoV-2 is unlikely to lead to asthma exacerbations.^39^^,^^40^ A plausible explanation for this are mechanistic differences between how a virus binds to cells in the respiratory tract between common respiratory viruses such as rhinoviruses and SARS-CoV-2. Rhinoviruses use ICAM-1 molecule (over-expressed within asthma patients with allergic airways), but SARS-CoV-2 uses angiotensin-converting enzyme 2 (ACE2) and transmembrane protease serine 2 (TMPRSS2) receptors.^40^ In fact, some studies have hypothesised that asthma may, instead, provide a protective effect against infection with SARS-CoV-2 since the expression of ACE2 receptors is decreased in patients with asthma due to use of inhaled corticosteroids.^39^

The key strengths of this study are the use of a large, country-wide dataset with over half a million asthma patients of whom over 100,000 developed an exacerbation, a long follow-up period (January 2016-October 2021) with 18 months during the pandemic, the use of previously validated algorithms for identifying asthma patients and ascertaining asthma exacerbation episodes, and the use of clinician-recorded, routinely collected primary care data from over 500 independent GP primary care practices with different clinical data recording systems covering a large geographic area. To our knowledge, this is the longest and one of the largest studies to assess the impact of the pandemic on asthma exacerbations. By restricting our analyses to only those who experienced at least one exacerbation episode during follow-up, we have excluded the following those: whose asthma resolved; who were earlier misdiagnosed with asthma during the cohort selection period, January 2010-December 2015; whose symptoms are so well-controlled that they never had any episode of exacerbation in the follow-up period (almost six years).

There are, however, six key limitations to note. First, despite being large and multi-centered, this was a descriptive study, and we cannot definitively identify causal factors that may explain the substantial reduction in exacerbation rates observed. This study has allowed us to hypothesise the likely mechanisms, but these now need further detailed investigation. Second, we were only able to access primary care dataset and any data records of patients self-referring to secondary care services were not directly accessible from hospital databases. However, we believe that any self-referred, asthma-related hospitalisation episodes that are not recorded in primary care will be rare. This is because all hospitalisations are accompanied by a discharge letter that gets sent to primary care via a NHS document system, and the majority get coded in primary care.^36^ In addition, primary care is likely the first and most frequent point of contact of patients with chronic conditions and any previous hospitalisation episode will likely end up being coded in the patient's primary care records. Third, this pandemic led to unprecedented strain on healthcare services, and many primary care practices reduced face-to-face consultations and moved to remote consultations.^1^ In this study, we have assumed that despite these changes, the GPs did not change their approach towards the use of Read codes in managing asthma patients. We believe that our assumption is highly likely to be valid since any ad-hoc changes towards the use of Read codes would likely have led to a non-uniform pattern in exacerbation rate change across different regions in England. However, the drastic reduction in exacerbations rates during the pandemic was uniformly consistent across all regions in England. Fourth, we were unable to investigate any potential impact of ethnicity and socioeconomic position on asthma exacerbations during the pandemic due to lack of data availability. Fifth, although we have speculated on what could be the most plausible causal factors that have led to such an unprecedented reduction in asthma exacerbations, additional explanations for the sustained reduction in asthma exacerbations such as sustained reduction in airway inflammation, and lifestyle changes (that reduces exposure to asthma exacerbation triggers) cannot be ruled out. Sixth, it was not possible to further stratify our cohort based on different asthma phenotypes to assess if the exacerbation rates are different during the follow-up period.

This study provides a clear and compelling demonstration that substantial reduction in asthma exacerbations is possible without additional pharmacological interventions (beyond what is currently available). Further research is now required to design and develop pragmatic non-pharmacological interventions that could reduce exposure to respiratory viruses and promote improved self-management to ensure that the low rate of asthma exacerbations persists beyond the COVID-19 pandemic. Clinical approaches to manage asthma patients should also be reconsidered such as redesign of waiting rooms in different clinical settings, and the use of remote consultations to mitigate the risks of exposure to other respiratory viruses.^2^ In summary, this large, national analysis has shown substantial reductions in asthma exacerbations across England over the first 18 months since lockdown. These reductions have been seen in all ages, both sexes, all sub-regions of England and during varying social restrictions. As such, these findings are unlikely to be adequately explained by changes in health-seeking behaviour, pandemic-related healthcare service disruption, or improvements in air quality. A reduction in exposure to common respiratory viruses due to pandemic-related, non-pharmacological, public health measures is likely the most plausible explanation. There is a need to identify acceptable and effective ways of reducing respiratory virus exposure in patients with asthma beyond the pandemic.

## Contributors

AS conceived this analysis and oversaw all aspects of the study. SAS with AS and JQ designed the study. SAS undertook all the analysis and wrote the first draft of the manuscript. All authors commented critically on the manuscript and approved the final version.

## Data availability statement

The data used in this study can be accessed from OPCRD (https://opcrd.co.uk/) subject to licensing agreement and independent evaluation of the scientific protocol. All the code used in this study will be made publicly available at GitHub (https://github.com/syedahmar/) and the associated metadata will also be made available via BREATHE.

## Declaration of interests

AS is a member of the Scottish Government Chief Medical Officer's COVID-19 Advisory Group and its Standing Committee on Pandemics, and NERVTAG's Risk Stratification Subgroup. All other authors declare no conflict of interest related to this work.

## Appendix Supplementary materials

Image, application 1
