# Supplementary material for: Impact of COVID-19 pandemic on asthma exacerbations: Retrospective cohort study of over 500,000 patients in a national English primary care database
Source: Lancet Reg Health Eur. 2022 Jun 15;19:100428. doi: 10.1016/j.lanepe.2022.100428 (PMC9213032; doi:10.1016/j.lanepe.2022.100428)
Supplement: Supplementary file 1 [file mmc1.docx]

**Impact of COVID-19 pandemic on asthma exacerbations: Retrospective cohort study of over 500,000 patients in a national English primary care database**

Contents

[SARS-CoV-2 Infection Levels During Follow-Up 2](#_Toc103759696)

[Illustration of Methodology to Compute Quarterly Rates 3](#_Toc103759697)

[Illustration of methodology adopted to Construct Control Time-Series 4](#_Toc103759698)

[Stratified Asthma Exacerbation Rates During Follow-Up 5](#_Toc103759699)

[Total Primary Care Appointments Across England During Follow-Up 8](#_Toc103759700)

[Sensitivity Analyses 9](#_Toc103759701)

[Read Codes Used 10](#_Toc103759702)

[Age and Sex Distribution of the Cohort 11](#_Toc103759703)

[Yearly Age-breakdown of Young Children (0-5) 12](#_Toc103759704)

[Literature Review 12](#_Toc103759705)

[Literature Review References 13](#_Toc103759706)

# SARS-CoV-2 Infection Levels During Follow-Up


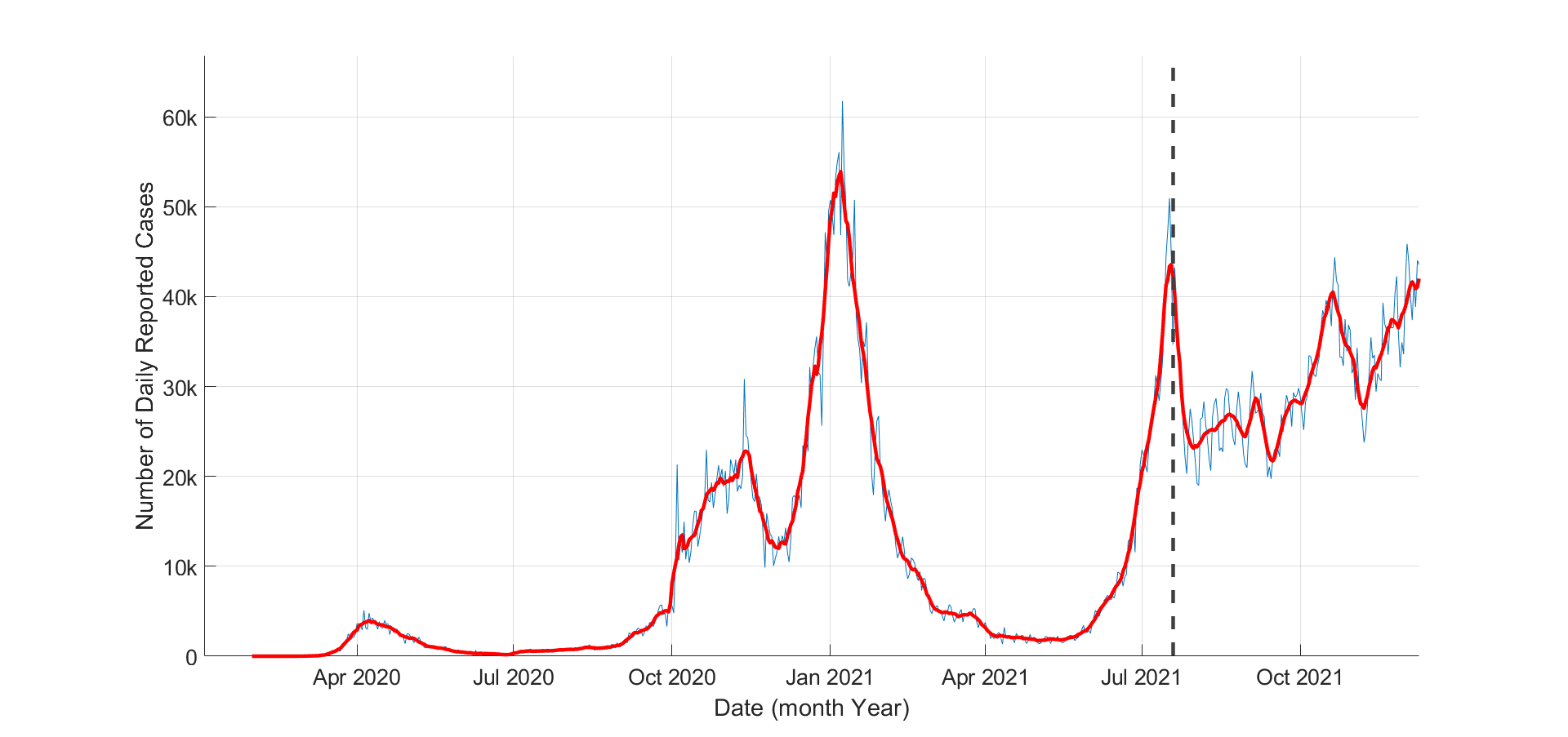


Figure S1: Total number of daily reported SARS-CoV-2 cases along with a 7-day rolling average mean. The dashed vertical line shows July 19, 2021, when most pandemic-related restrictions were lifted across England.

# Illustration of Methodology to Compute Quarterly Rates


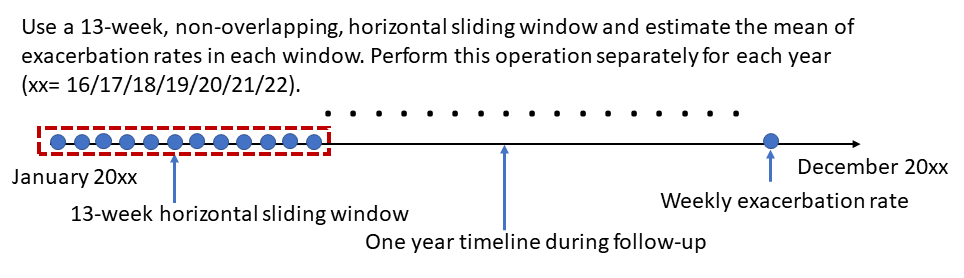


Figure S2: Illustration of methodology adopted to estimate quarterly exacerbation rates with a 13-point, non-overlapping, horizontal sliding window

# Illustration of methodology adopted to Construct Control Time-Series


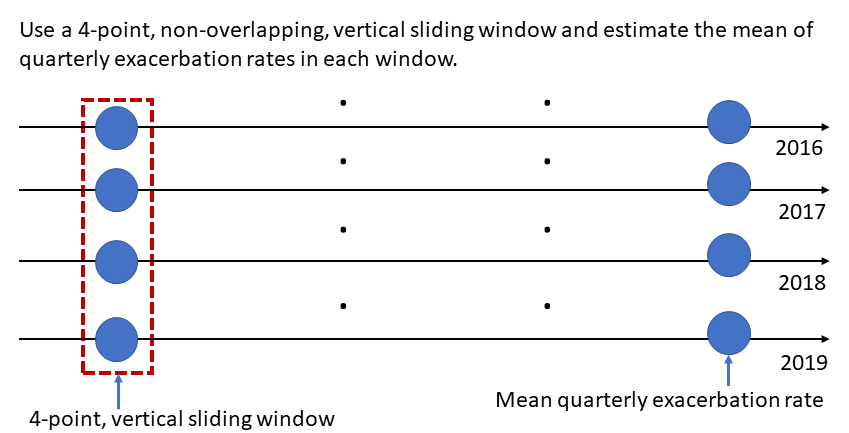


Figure S3: Illustration of methodology adopted to construct a control time-series with a 4-point, non-overlapping, vertical sliding window

# Stratified Asthma Exacerbation Rates During Follow-Up


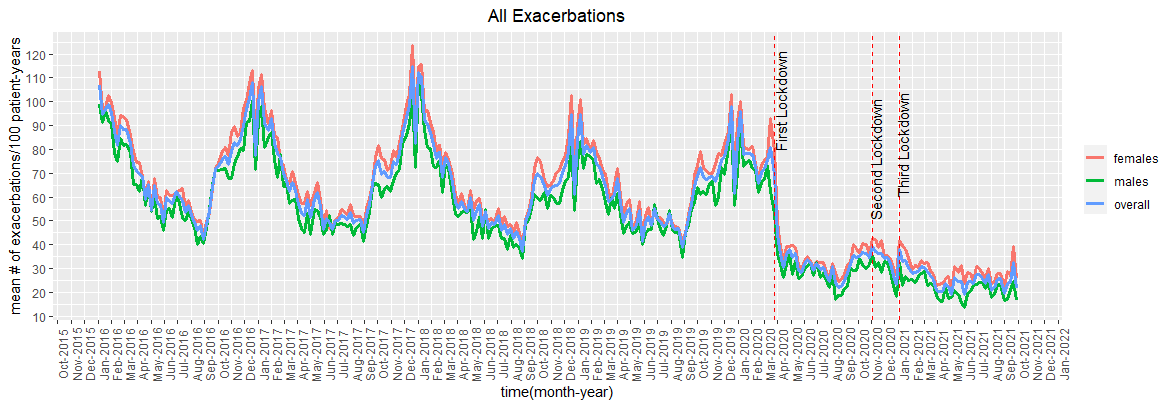


Figure S4: Mean exacerbations rate (total number of exacerbation episodes per 100 patient-years) stratified by sex during the follow-up period (January 2016 – October 2021)


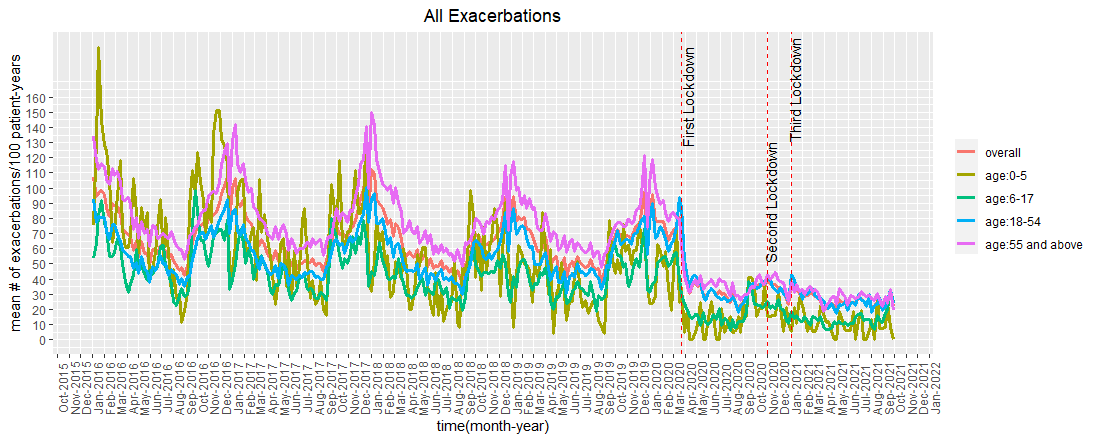


Figure S5: Mean exacerbations rate (total number of exacerbation episodes per 100 patient-years) stratified by age group during the follow-up period (January 2016 – October 2021)


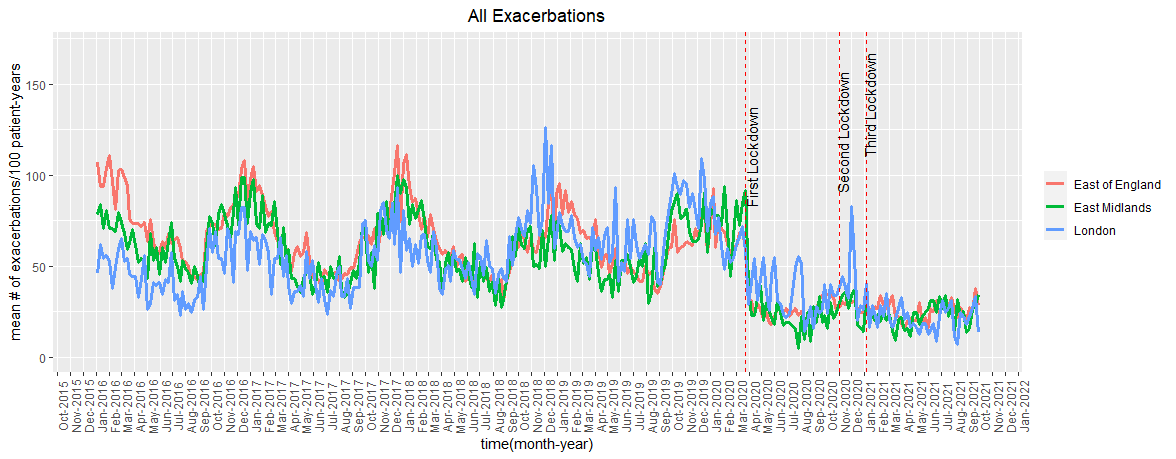


Figure S6: Mean exacerbations rate (total number of exacerbation episodes per 100 patient-years) stratified by English region (East of England, East Midlands, London) during the follow-up period (January 2016 – October 2021)


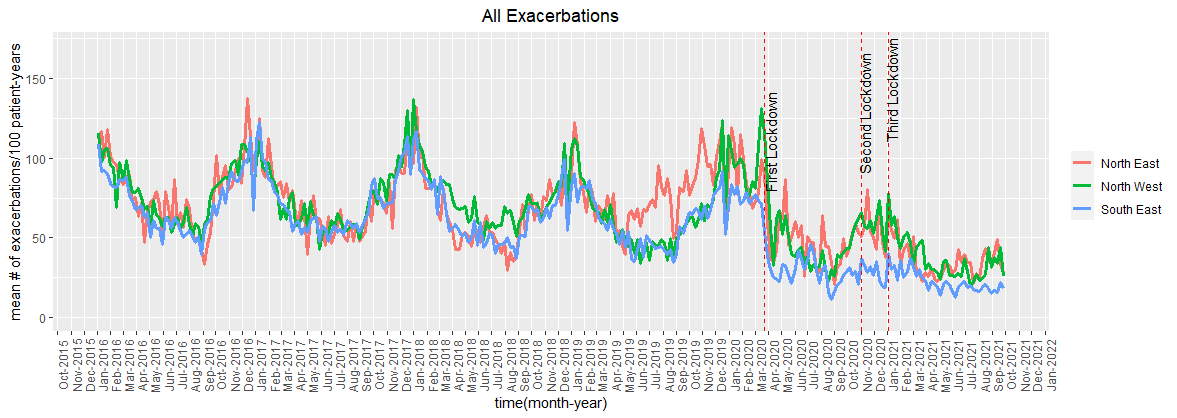


Figure S7: Mean exacerbations rate (total number of exacerbation episodes per 100 patient-years) stratified by English region (North East, North West, South East) during the follow-up period (January 2016 – October 2021)


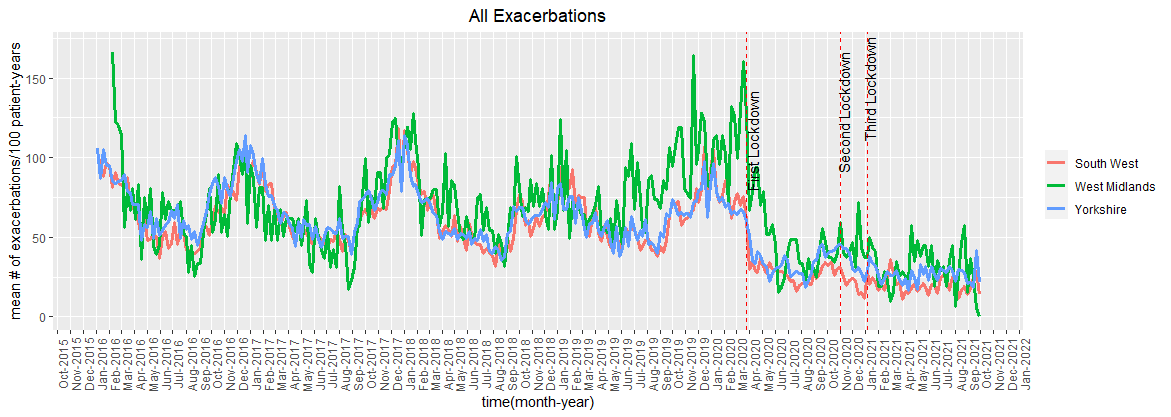


Figure S8: Mean exacerbations rate (total number of exacerbation episodes per 100 patient-years) stratified by English region (South West, West Midlands, Yorkshire) during the follow-up period (January 2016 – October 2021)

**
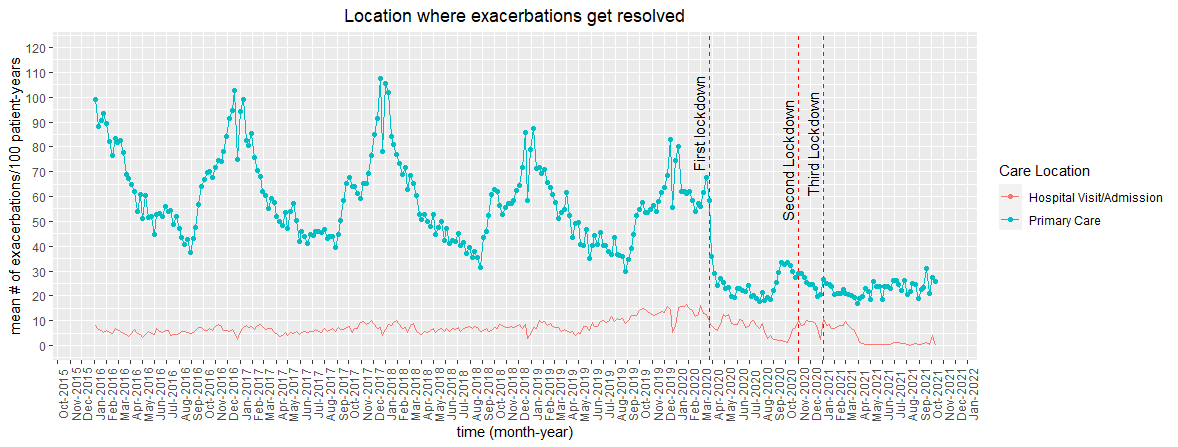
**

Figure S9: Mean exacerbations rate (total number of exacerbation episodes per 100 patient-years) stratified by location where the exacerbation gets resolved (primary care or hospital attendance/admission) during the follow-up period (January 2016 – October 2021)

# Total Primary Care Appointments Across England During Follow-Up


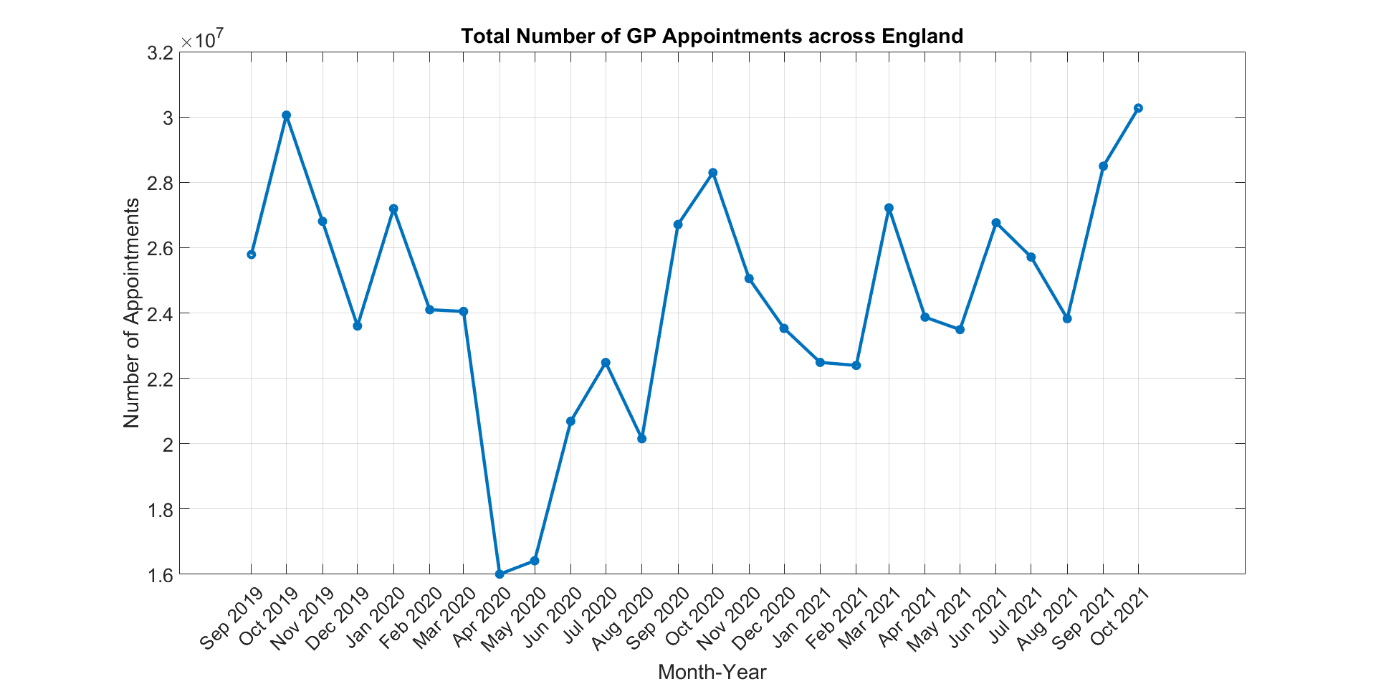


Figure S11: Total monthly GP appointments in England from September 2019 - October 2021

# Sensitivity Analyses


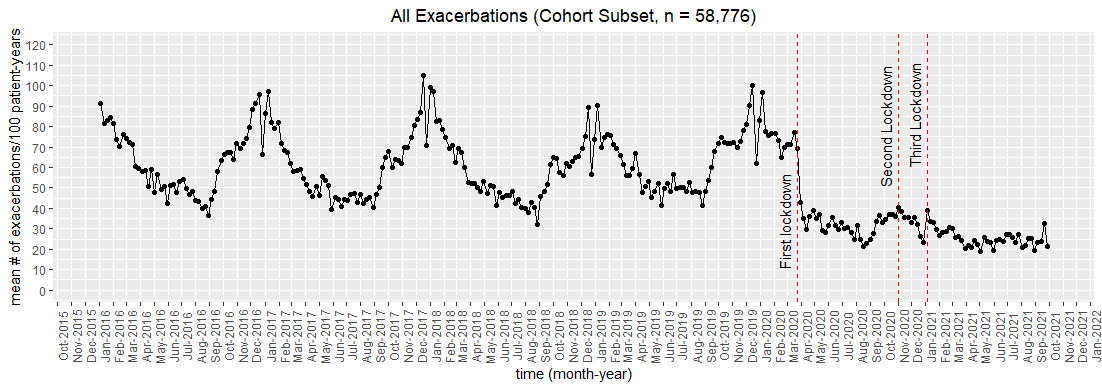


Figure S12: Mean exacerbation rates during the follow-up after restricting the cohort to those patients who remained in the study until at least the last quarter


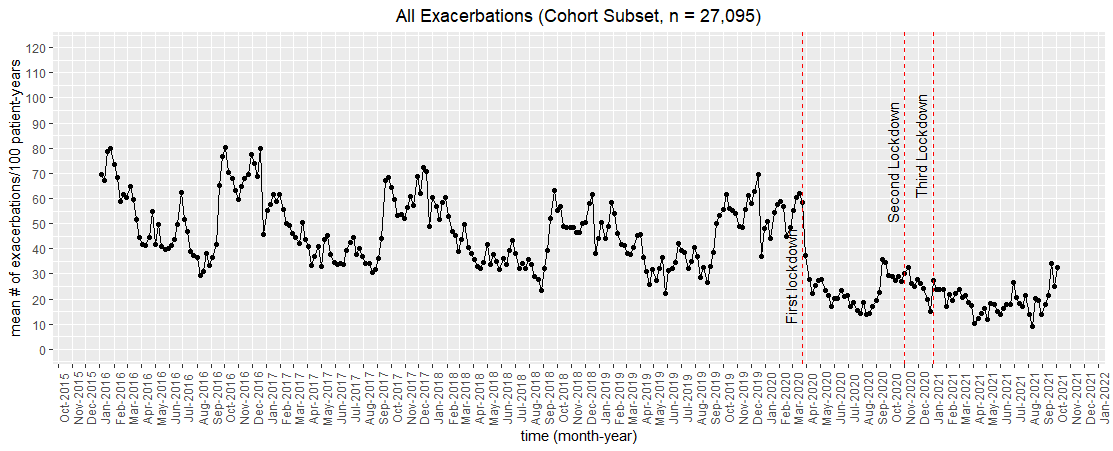


Figure S13: Mean exacerbation rates during the follow-up after restricting the cohort to those aged 34 or under at the follow-up start date

# Read Codes Used

**Asthma exacerbation**

Xa1hD","Xafdy","Xafdz","Xafdj","XE0YW","XM0s2","X101y","X1022","H333.","H3301","H3311","H33z0","H33z1

**Asthma hospitalization or Accident & Emergency visit**

"663d".,"8H2P.","663m."

**Oral corticosteroids**

"fe6..","fe3..","fe31.","fe32.","fe33.","fe36.","fe37.","fe3A.","fe3B.","fe3C.","fe3r.","fe3s.","fe3u.","fe4..","fe41.","fe42.","fe43.","fe44.","fe45.","fe4e.","fe4f.","fe4g.","fe4h.","fe5..","fe51.","fe52.","fe53.","fe5f.","fe5m.","fe5n.","fe5o.","fe5p.","fe61.","fe62.","fe64.","fe65.","fe66.","fe67.","fe68.","fe69.","fe6a.","fe6c.","fe6d.","fe6e.","fe6f.","fe6g.","fe6h.","fe6i.","fe6j.","fe6k.","fe6l.","fe6m.","fe6n.,"fe6o.","fe6p.","fe6q.","fe6r.","fe6s.","fe6t.","fe6v.","fe6w.","fe6z.","fe7..","fe71.","fe72.","fe73.","fe74.","fe75.","fe76.","fe77.","fe78.","fe79.","fe7x.","fe7y.","fe7z.","x00yP","x01Mh","x01Na","x01Nb","fe11.","fe12.","fe1x.","fe1y.","fe21.","fe22.","fe23.","fe24.","fe25.","fe26.","x01MW"

**Evidence of lower respiratory review**

“XE1P1”,”XE2Nb”,”XE2b5”,”XM0rv”,”XM0s2”,”XM1Bs”,”XM1QV”,”XM1QX”,”XSCET”,”Xa0Y7”,”Xa0lY”,”Xa0lZ”,”Xa1hD”,”Xa35l”,”Xa7nL”,”Xa7nM”,”Xa7nN”,”Xa7nP”,”Xa7nT”,”Xa7nU”,”Xa8Hn”,”Xa97Z”,”Xa9zf”,”XaBE9”,”XaBM8”,”XaBfJ”,”XaDcD”,”XaDcV”,”XaDsa”,”XaDtB”,”XaDtP”,”XaDth”,”XaDtl”,”XaDvK”,”XaDvL”,”XaEFy”,”XaEFz”,”XaEIV”,”XaEIW”,”XaEIY”,”XaF6d”,”XaF6e”,”XaFrU”,”XaFrV”,”XaFrW”,”XaFrX”,”XaIIW”,”XaIIX”,”XaIIY”,”XaIIZ”,”XaIND”,”XaINZ”,”XaINa”,”XaINb”,”XaINc”,”XaINd”,”XaINf”,”XaINg”,”XaINh”,”XaIQ2”,”XaIQ3”,”XaIQ4”,”XaIQD”,”XaIQE”,”XaIQg”,”XaIR3”,”XaIUi”,”XaIUl”,”XaIUm”,”XaIUn”,”XaIUo”,”XaIeq”,”XaIer”,”XaIet”,”XaIfK”,”XaIoE”,”XaIu5”,”XaIu6”,”XaIuG”,”XaIww”,”XaIxQ”,”XaIxR”,”XaIxU”,”XaIxV”,”XaJ3K”,”XaJ9B”,”XaJ9C”,”XaJ9D”,”XaJ9E”,”XaJEl”,”XaJFG”,”XaKdk”,”XaLIm”,”XaLIn”,”XaLIr”,”XaLJO”,”XaLJS”,”XaLJT”,”XaLJU”,”XaLPE”,”XaMeu”,”XaN4a”,”XaNKw”,”XaObi”,”XaObj”,”XaObk”,”XaObl”,”XaObm”,”XaPpI”,”XaQHq”,”XaQig”,”XaQih”,”XaQmR”,”XaQmS”,”XaQmT”,”XaQmU”,”XaREU”,”XaRFi”,”XaRFj”,”XaRFk”,”XaRFl”,”XaVx3”,”XaX3n”,”XaXCa”,”XaXCb”,”XaXZm”,”XaXZp”,”XaXZs”,”XaXZu”,”XaXZx”,”XaXa0”,”XaXeg”,”XaY2V”,”XaYYt”,”XaYYu”,”XaYZB”,”XaYZh”,”XaYb8”,”XaYby”,”XaYja”,”XaYmL”,”XaYpC”,”XaYpF”,”XaZ1k”,”XaZ1l”,”XaZd1”,”XaZyl”,”XaZyt”,”XaZyu”,”XaZz1”,”Xaa7B”,”Xaa7C”,”Xaa7Q”,”Xaafd”,”XaahD”,”Xab2f”,”Xab4X”,”ZV19L”,”ZV725”,”ZV74B”,”ZV7B3”,””c74..”

**Additional non-asthma specific codes for hospitalisation**

“8HJJ.00”,”9b8D.00”,”8HX..00”,”8HX2.00”,”8Hd..00”,”8H16.00”,”8H2R.00”,”8H29.00”,”8H2D.00”,”8H2J.00”,”8H24.00”,”8H26.00”,”8H2H.00”,”8H2Z.00”,”8H21.00”,”8H2E.00”,”8H2N.00”,”8H27.00”,”8H2B.00”,”8H2K.00”,”8H28.00”,”8H25.00”,”8H2I.00”,”8H23.00”,”8H2L.00”,”8H2G.00”,”8H2M.00”,”8H2C.00”,”8H22.00”,”8H1..11”,”8H15.00”,”8H1Z.00”,”8H1..00”,”8H14.00”,”8H13.00”,”8H12.00”,”8H2A.00”,”8H2F.00”,”SP31100”,”663m.00”,”66Yd.00”,”9b8B.00”,”9498”,”8HG..11”,”9451”,”L398400”,”8HG..00”,”ZLD2G11”,”ZLD2100”,”ZLD2H11”,”ZLD2H00”,”ZLD2111”,”ZLD2G00”,”ZLD2I11”,”ZLD2I00”,”ZLE1.00”,”ZLE5111”,”ZLE5100”,”ZLE1.11”,”ZLF2.00”,”ZLE5.00”,”ZLE5200”,”ZLG6.00”,”ZLG6100”,”ZLG6400”,”ZLG6500”,”8HE8.00”,”8HE..00”,”8HE2.00”,”8HN..00”,”7A10000”,”7A15000”,”7A12000”,”7A41400”,”7A47800”,”7A47200”,”7A47000”,”7A47600”,”7A47400”,”7A47C00”,”7A41000”,”7A41200”,”7A15300”,”7A41600”,”7A47B00”,”7A47300”,”7A47700”,”7A47100”,”7A47D00”,”8H23000”,”7A11000”,”7A11200”,”7A45300”,”7A13400”,”7A13000”,”7A45200”,”7A45000”,”7A40000”,”7A13300”,”7A45700”,”7A45D00”,”7A13100”,”66Ye.00”,”8H2P.00”,”7700.11”,”7700300”,”663d.00”,”7F13300”,”7700100”,”7700000”,”7700”,”7700z00”,”7700200”,”7A41211”,”8H2..00”,”8712”,”7M30000”,”7A13.11”,”7A45.15”,”7A45.00”,”7A45.14”,”7A13.00”,”7A13z00”,”7A13y00”,”8H2T.00”,”7700400”,”9239”,”9b0K.00”,”945Z.00”,”945..00”,”13F8.11”,”9b0L.00”,”13F8.00”,”9R6..00”,”7936500”,”8HN1.00”,”8HNA.00”,”8HNB.00”,”8HNC.00”,”8HND.00”,”8HNE.00”,”8HN2.00”,”8HN3.00”,”8HN4.00”,”8HN5.00”,”8HN6.00”,”8HN7.00”,”8HN8.00”,”8HN9.00”,”8HN0.00”,”8HNZ.00”,”8CO..00”,”Z177800”,”8A8..00”,”8Hb..00”,”8HM4.00”,”8HMB.00”,”8HM3.00”,”8HME.00”,”8HMJ.00”,”8HM8.00”,”8HM1.00”,”8HMG.00”,”8HM7.00”,”8HMO.00”,”8HM5.00”,”8HM6.00”,”8HML.00”,”8HMN.00”,”8HMD.00”,”8HMH.00”,”8HMQ.00”,”8HMK.00”,”8HMM.00”,”8HM2.00”,”8HMP.00”,”8HM9.00”,”8HMC.00”,”8HMA.00”,”8HMR.00”,”8HM..00”,”8HMZ.00”,”8HMF.00”,”13F8100”,”13FS.00”,”66Yi.00”,”8H32.00”,”7701.11”,”8H3E.00”,”8H3V.00”,”8H3T.00”,”8H3I.00”,”8H3O.00”,”8H39.00”,”8H3B.00”,”8H3M.00”,”8H31.00”,”8H3..00”,”8H36.00”,”8H3J.00”,”8H3S.00”,”8H3C.00”,”8H3G.00”,”8H3U.00”,”8H3D.00”,”8H3A.00”,”8H3N.00”,”8H38.00”,”8H3Q.00”,”8H3L.00”,”8H3R.00”,”8H3P.00”,”8H3H.00”,”8H37.00”,”8H3F.00”,”8H3K.00”,”7A47.11”,”7A47.12”,”7A47.13”,”7A47.16”,”7A47.00”,”7A47z00”,”7A47y00”,”7A47.14”,”7A15.00”,”7A47.15”,”8H3Z.00”,”7700y00”,”7259y00”,”7B07y00”,”7513y00”,”9b8C.00”,”949B.00”,”9495”,”9144”,”9N19.11”,”7B07.00”,”7B07z00”,”T772.00”,”9b8A.00”,”8HLP.00”,”8HKP.00”,”8HJI.00”,”8HC1.00”,”8H63.00”,”ZL51.11”,”8HC3.00”,”8H7a.00”,”8HD..00”,”8HC..00”,”8HCZ.00”,”ZL56.11”,”ZL51.00”,”ZL56100”,”ZL51.13”,”8HTF.00”,”ZL56.00”,”ZL56211”,”ZL56200”,”8HX1.00”,”9H4..00”,”9H5..00”,”ZL96.11”,”ZL91.00”,”ZL96111”,”ZL96100”,”ZL91.12”,”ZL96211”,”ZL96200”,”ZL9GQ00”,”9N04.00”,”9N1y300”,”9N19.00”,”9N1B.00”,”8HJ..00”,”8HJZ.00”,”8HF..12”,”7A13411”,”ZL16.11”,”ZL11.00”,”ZL16111”,”ZL16100”,”ZL11.12”,”ZL16.00”,”ZL16211”,”ZL16200”,”8HX0.00”,”8Ha..00”,”7A11211”,”ZV29300”,”ZV29211”,”ZV29200”

**Specific asthma codes previously validated to identify asthma patients in primary care**

“H33..”,”663..”,”H333.”,”H33z1”,”H33z0”,”H33..”,”H330.”,”663V1”,”663V3”,”663V0”,”H331.”,”H33z.”,”H33zz”,”H33z0”,”H331.”,”H3300”,”H3120”,”173A.”,”H3301”,”8H2P.”,”H330.”,”663P.”,”663U.”,”663N.”,”H330.”,”H33z1”,”9OJA.”,”663y.”,”66Y5.”,”66Y9.”,”66YJ.”,”8B3j.”,”663j.”,”1O2..”,”H33z2”,”663V.”,”663V2”,”663N2”,”66YK.”,”H3300”,”H330.”,”H33zz”,”8795”,”8794”,”66YA.”,”8796”,”H3311”,”66YQ.”,”663p.”,”663n.”,”9OJA.”,”8798”,”8797”,”H33zz”,”173c.”,”663d.”,”8791”,”663u.”,”663e.”,”8CR0.”,”663s.”,”663v.”,”663f.”,”663e1”,”663e0”,”H3301”,”H3310”,”66YR.”,”663N0”,”66YP.”,”663t.”,”663O0”,”663w.”,”663x.”,”663N1”,”H35y7”,”663r.”,”H334.”,”1780”,”66YC.”,”663q.”,”H331z”,”H330z”,”9OJ1.”,”663m.”,”H47y0”,”H3311”,”173d.”,”H35y6”,”388t.”,”38DL.”,”8CMA0”,”679J0”,”38DT.”,”9NNX.”,”679J1”,”66Yp.”,”38DV.”,”1787”,”1781”,”66Yr.”,”66Yq.”,”1789”,”663P0”,”178B.”,”663P1”,”1783”,”679J2”,”1786”,”66Ys.”,”388t0”,”1788”,”178A.”,”1785”,”1784”,”1782”,”663P2”,”661N1”,”661M1”,”H335.”,”66Yu.”

# Age and Sex Distribution of the Cohort

Table 1: Age and sex breakdown of the asthma cohort in the study

| **Age Group** | **Males, n (%)** | **Females, n (%)** | **Overall, n** |
| --- | --- | --- | --- |
| **0-5** | 884 (65.1) | 473 (34.9) | 1,357 |
| **6-17** | 5,293 (57.1) | 4,455 (42.9) | 10,378 |
| **18-54** | 15,724 (36.2) | 27,749 (63.8) | 43,473 |
| **55+** | 16,391 (37.5) | 27,313 (62.5) | 43,704 |
| **All (0+)** | 38,922 (39.4) | 59,990 (60.6) | 98,912 |

# Yearly Age-breakdown of Young Children (0-5)

| **Age** | **Number** |
| --- | --- |
| 0-5 | 1,375 |
| 0-1 | 9 (0.7%) |
| 1-2 | 55 (4.0%) |
| 2-3 | 201 (14.6%) |
| 3-4 | 489 (35.6%) |
| 4-5 | 621 (45.2%) |

# Literature Review

Table S1 The 20 identified studies after searching for relevant studies on medRxiv and PubMed that reported on patterns of asthma exacerbations during the pandemic. The data source (routinely collected or self-reported), follow-up duration, location, study setting (hospital, or primary care), and study population type (children/adults/everyone) are provided.

| Reference | Duration | Location | Study Setting | Additional Information |
| --- | --- | --- | --- | --- |
| Quintyne et al. (1) | January 2018-February 2021 | Dublin, Ireland | Hospital | - |
| Fan et al. (2) | February to June 2020 | Guangzhou, China (single hospital) | Hospital | Children only |
| Sykes et al. (3) | March 23 - June 1, 2020 (compared with 2018,2019) | Hull Trust (2 regional hospitals) | Hospital |  |
| Pepper et al. (4) | July 2018-July 2020 | Single Hospital, US | Hospital | Children only |
| Mansfield et al. (5) | 2017-July 2020 | UK-Wide, (CPRD Aurum) | Primary Care | 11 years or older only |
| Yamaguchi et al. (6) | 2011-2020 | Single Hospital, Kobe City, Japan | Hospital | Children only (<16 years) |
| Alsulaiman et al. (7) | 2018-2020 | Two hospitals, Jordan | Hospital | Children only |
| Golan-Tripto et al. (8) | 2019-June 2020 | Single Hospital, Israel | Hospital | Children only (2-18 years) |
| Shah et al. (9) | 2016-August 2020 | Country-Wide, England | Primary-Care |  |
| Sigala et al. (10) | 2019-May 2020 | Athens, Greece | Hospital |  |
| Salciccioli et al. (11) | 2019-2020 (Quarters1 and 2) | US-wide recruitment (from both primary and secondary care) | Trial with recruitment from both; self-reported | Adults only |
| Chan et al. (12) | 2016-April 2020 | Country-Wide, Hong Kong | Hospital | Adults only |
| Caruso et al. (13) | 30 March 2020-30 April 2020 | Italy (100 patients) | Recruited from patients who referred to their center; self-reported |  |
| Taytard et al. (14) | 2019-2020 | Single Hospital, Paris, France | Hospital | Children only |
| Bover-Bauza et al. (15) | 14 March -15 April (2019-2020) | Single Hospital, Spain | Hospital | Children only |
| Boer et al. (16) | Until 1 July 2020 | From a Randomised Controlled Trial in Netherlands | Trial; self-reported verified with hospital data |  |
| Papadopoulos et al. (17) | Short-term, at least 2 months after pandemic onset | Global, PeARL study from 15 countries | Trial; self-reported | Children only |
| Bun et al. (18) | July 1, 2018-June 30, 2020 | Country-wide, Japan | Hospital | Children (≤15 years) |
| Davies et al. (19) | Until 03 May, 2020 | Country-wide, Scotland and Wales | Hospital |  |
| Wee et al. (20) | Until November 2020 | Singapore | Hospital | Children only |

# Literature Review References

1. Quintyne KI, Kelly C, Sheridan A, Kenny P, O’Dwyer M. COVID-19 transport restrictions in Ireland: impact on air quality and respiratory hospital admissions. Public Health. 2021;198:156–60.

2. Fan H, He C, Yin G, Qin Y, Jiang N, Lu G, et al. Frequency of asthma exacerbation in children during the coronavirus disease pandemic with strict mitigative countermeasures. Pediatr Pulmonol. 2021;56(6):1455–63.

3. Sykes DL, Faruqi S, Holdsworth L, Crooks MG. Impact of COVID-19 on COPD and asthma admissions, and the pandemic from a patient’s perspective. ERJ Open Res. 2021;7(1).

4. Pepper MP, Leva E, Trivedy P, Luckey J, Baker MD. Analysis of pediatric emergency department patient volume trends during the COVID-19 pandemic. Medicine (Baltimore). 2021;100(27).

5. Mansfield KE, Mathur R, Tazare J, Henderson AD, Mulick AR, Carreira H, et al. Indirect acute effects of the COVID-19 pandemic on physical and mental health in the UK: a population-based study. Lancet Digit Heal. 2021;3(4):e217–30.

6. Yamaguchi H, Nozu K, Ishiko S, Kondo A, Ninchoji T, Takeda H, et al. Impact of the state of emergency during the COVID-19 pandemic in 2020 on asthma exacerbations among children in Kobe City, Japan. Int J Environ Res Public Health. 2021;18(21):11407.

7. Alsulaiman JW, Kheirallah KA, Ajlony M, Al‐Tamimi T, Khasawneh RA, Al‐Natour L. Pediatric Asthma Exacerbation Admissions and Stringency of Non‐Pharmaceutical Interventions; Results from a Developing Country. Int J Clin Pract. 2021;e14423.

8. Golan‐Tripto I, Arwas N, Maimon MS, Bari R, Aviram M, Gatt D, et al. The effect of the COVID‐19 lockdown on children with asthma‐related symptoms: A tertiary care center experience. Pediatr Pulmonol. 2021;

9. Shah SA, Quint JK, Nwaru BI, Sheikh A. Impact of COVID-19 national lockdown on asthma exacerbations: interrupted time-series analysis of English primary care data. Thorax. 2021;

10. Sigala I, Giannakas T, Giannakoulis V, Zervas E, Mprinia A, Gianniou N, et al. Effect of COVID-19-related Lockdown οn Hospital Admissions for Asthma and COPD Exacerbations: Associations with Air Pollution and Patient Characteristics. 2021;

11. Salciccioli JD, She L, Tulchinsky A, Rockhold F, Cardet JC, Israel E. Effect of COVID-19 on asthma exacerbation. J Allergy Clin Immunol Pract. 2021;

12. Chan KPF, Kwok W-C, Ma T-F, Hui C-H, Tam TC-C, Wang JK-L, et al. Territory-wide Study on Hospital Admissions for Asthma exacerbation in COVID-19 Pandemic. Ann Am Thorac Soc. 2021;(ja).

13. Caruso C, Colantuono S, Urbani S, Heffler E, Canonica GW, Andriollo G, et al. Real-life survey on severe asthma patients during COVID-19 lockdown in Italy. Expert Rev Respir Med. 2021;1–4.

14. Taytard J, Coquelin F, Beydon N. Improvement in Asthma Symptoms and Pulmonary Function in Children After SARS-CoV-2 Outbreak. Front Pediatr. 2021;9.

15. Bover-Bauza C, Gomila MAR, Pérez DD, Pons ARM, Sánchez JAG, Peña-Zarza JA, et al. The Impact of the SARS-CoV-2 Pandemic on the Emergency Department and Management of the Pediatric Asthmatic Patient. J Asthma Allergy. 2021;14:101.

16. De Boer G, Braunstahl G-J, Hendriks R, Tramper-Stranders G. Asthma exacerbation prevalence during the COVID-19 lockdown in a moderate-severe asthma cohort. BMJ open Respir Res. 2021;8(1):e000758.

17. Papadopoulos NG, Mathioudakis AG, Custovic A, Deschildre A, Phipatanakul W, Wong G, et al. Childhood asthma outcomes during the COVID‐19 pandemic: Findings from the PeARL multinational cohort. Allergy. 2021;

18. Bun S, Kishimoto K, Shin J, Takada D, Morishita T, Kunisawa S, et al. Impact of the COVID-19 Pandemic on Infant and Pediatric Asthma: A Multi-Center Survey Using an Administrative Database in Japan (preprint). 2020;

19. Davies GA, Alsallakh MA, Sivakumaran S, Vasileiou E, Lyons RA, Robertson C, et al. Impact of COVID-19 lockdown on emergency asthma admissions and deaths: national interrupted time series analyses for Scotland and Wales. Thorax. 2021;

20. Wee LE, Conceicao EP, Tan JY, Sim JXY, Venkatachalam I. Reduction in asthma admissions during the COVID-19 pandemic: consequence of public health measures in Singapore. Eur Respir J. 2021;57(4).
